# Therapeutic and diagnostic applications of exosomal circRNAs in breast cancer

**DOI:** 10.1007/s10142-023-01083-3

**Published:** 2023-05-27

**Authors:** Mohanraj Gopikrishnan, Hephzibah Cathryn R, Gnanasambandan R, Hossam M. Ashour, Gianfranco Pintus, Mohamed Hammad, Manoj Kumar Kashyap, George Priya Doss C, Hatem Zayed

**Affiliations:** 1 Laboratory of Integrative Genomics, Department of Integrative Biology, School of BioSciences and Technology, Vellore Institute of Technology (VIT), Vellore, 632014 Tamil Nadu India; 2 Department of Integrative Biology, College of Arts and Sciences, University of South Florida, St. Petersburg, Florida 33701 USA; 3 Department of Biomedical Sciences, University of Sassari, 07100 Sassari, Italy; 4 Department of Stem Cell Biology and Regenerative Medicine, City of Hope Beckman Research Institute, Duarte, California USA; 5 Amity Stem Cell Institute, Amity Medical School, Amity University Haryana, Manesar (Gurugram), Panchgaon, Haryana (HR) 122413 India; 6 Clinical Biosamples & Research Services (CBRS), Noida, Uttar Pradesh 201301 India; 7 Department of Biomedical Sciences, College of Health Sciences, QU Health, Qatar University, 2713 Doha, Qatar

**Keywords:** Breast cancer, circRNA, miRNA sponges, circRNA biomarkers, Exosomal circRNAs, Tumor-suppressive circRNAs

## Abstract

Circular RNAs (circRNAs) are regulatory elements that are involved in orchestrating gene expression and protein functions and are implicated in various biological processes including cancer. Notably, breast cancer has a significant mortality rate and is one of the most common malignancies in women. CircRNAs have been demonstrated to contribute to the pathogenesis of breast cancer including its initiation, progression, metastasis, and resistance to drugs. By acting as miRNA sponges, circRNAs can indirectly influence gene expression by disrupting miRNA regulation of their target genes, ultimately altering the course of cancer development and progression. Additionally, circRNAs can interact with proteins and modulate their functions including signaling pathways involved in the initiation and development of cancer. Recently, circRNAs can encode peptides that play a role in the pathophysiology of breast cancer and other diseases and their potential as diagnostic biomarkers and therapeutic targets for various cancers including breast cancer. CircRNAs possess biomarkers that differentiate, such as stability, specificity, and sensitivity, and can be detected in several biological specimens such as blood, saliva, and urine. Moreover, circRNAs play an important role in various cellular processes including cell proliferation, differentiation, and apoptosis, all of which are integral factors in the development and progression of cancer. This review synthesizes the functions of circRNAs in breast cancer, scrutinizing their contributions to the onset and evolution of the disease through their interactions with exosomes and cancer-related intracellular pathways. It also delves into the potential use of circRNA as a biomarker and therapeutic target against breast cancer. It discusses various databases and online tools that offer crucial circRNA information and regulatory networks. Lastly, the challenges and prospects of utilizing circRNAs in clinical settings associated with breast cancer are explored.

## Introduction

About 2% of DNA transcripts in the genome of eukaryotic organisms can be translated into functional proteins (Djebali et al. 2012). The remaining 98% of the non-coding RNA (ncRNA) were considered irrelevant due to their non-coding ability for biologically active proteins (Mattick 2001; Statello et al. 2021). Interestingly, in the 1990s, evidence for the involvement of small ncRNA and microRNA (miRNA) in several diseases emerged (Ghosh et al. 2023; Dehaini et al. 2019; Giordo et al. 2021, 2022; Hu et al. 2022; Posadino et al. 2022; Wehbe et al. 2019). These raise the interest in other forms of ncRNA such as long non-coding RNA (lncRNA) (Li et al. 2023), and the circular RNA (circRNA). CircRNAs are ncRNAs generated by back splicing, a non-canonical splicing process in which a downstream splicing donor site is chemically linked to an upstream splicing-acceptor site (Barrett and Salzman 2016). CircRNAs are ssRNA molecules that covalently binds to the end of an upstream 5’ acceptor site and a downstream 3’ donor site to generate circular form derived from splicing of exons from different mRNAs (Eger et al. 2018). Due to their resistance to exonuclease, circRNAs have a longer half-life (> 48 h) than regular RNA (Jeck and Sharpless 2014). CircRNAs can be divided into three categories: (i) exonic circular RNAs (EciRNAs) that contain only exons, are mostly cytoplasmic, and may contain miRNA elements that act as miRNA sponges, (ii) exon–intron circular RNA (EIciRNAs) that are circularized with introns that hold on between exons, and (iii) intronic circular RNA (ciRNAs) that are composed of introns. Most circRNAs are generated by exon hopping during pre-messenger RNA (pre-mRNA) transcription (Kelly et al. 2015), which generates an exon-containing lariat structure that is subsequently internally spliced to release introns and form EciRNAs (Eger et al. 2018). CircRNAs can regulate a variety of biological processes including tumor cell growth, apoptosis, inhibition of cell death, invasion and migration, and angiogenesis (Li et al. 2023; Ghosh et al. 2023; Loganathan and Doss 2023). Further, circRNAs interact with and modulate several cancer-associated intracellular pathways such as Wnt/beta-catenin, PIK3/AKT, and MAPK/ERK signaling (Solé and Lawrie 2020). CircRNAs play an important role in cancer pathogenesis by regulating cancer-associated genes by competing with miRNAs (Memczak et al. 2013), which are directly translated into functional proteins and act as a reservoir for miRNAs forming fusion circRNAs (fcircRNAs). According to World Health Organization (WHO), in 2020 alone, 2.26 million cases of breast cancer were reported. In India, it is estimated to be 13.5%. The mortality rate was 10.6% with a cumulative risk of 2.81 (Sung et al. 2021). In 2022, it is predicted to be 43,250 cases in women and 530 cases in men (Cancer Facts and Figs. 2022| American Cancer Society, 2022, and https://www.bcrf.org/breast-cancer-statistics-and-resources). Metastatic breast cancer is still one of the major causes of mortality despite substantial improvements in detection and treatment options (Lei et al. 2021).

Our understanding of the genetic modifications involved in BC tumorigenesis will help in the identification of associated molecular pathways and the use of genetic markers for prognosis and targeted treatment (Hu et al. 2009). Circular RNAs that are covalently closed are one of the recently identified genetic components that may be involved in BC. These molecules were recently discovered to be a distinct new class of competing endogenous RNAs (ceRNAs), a subset of long noncoding RNAs with no cap and a poly A tail in the 5′ and 3′ of their structure (Hansen et al. 2013). CircRNAs do not have any free 5′ and 3′ ends in their structure, making them resistant to exoribonucleases; hence, their transcripts are very stable and conserved. CircRNA dysregulation is also associated with several diseases including cancer, neurological problems, cardiovascular, and rheumatic diseases (He et al. 2021; Almaghrbi et al. 2023; Abbas et al. 2023). Disease-linked variations in the expression of many circRNAs may make them potential biomarkers for several complex human diseases. CircRNAs are abundant in biological specimens such as blood, fluid (Memczak et al. 2015), urine (Jacky Lam and Dennis Lo 2019), and exosomes. As per findings from next-generation RNA sequencing, more than 20% of protein-coding genes in the examined cells and tissues might produce circRNAs. High-throughput sequencing studies show that the human brain, saliva, exosomes, and peripheral blood contain large amounts of circRNAs (Jahani et al. 2020). There are a handful of databases on circRNAs, such as exoRBase (http://www.exorbase.org/) and MiOncoCirc (https://mioncocirc.github.io/), which compile circRNAs derived from human body fluids (Li et al. 2018) and clinical cancer samples (Zhao et al. 2020). The following criteria were used during our study: research showing and characterizing circRNA expression in various breast cancer types and stages, investigations on the association between circRNA and breast cancer human samples or cell lines and discussion of their potential use as a biomarker for early diagnosis, studies examining circRNA expression using microarray, studies discussing the relationship between circRNA and breast cancer survival, studies debating the relationship between circRNA and chemotherapy-resistant metastatic breast cancer, and studies specifically describing the roles of circRNA in the ceRNA network and its role in breast and other cancers.

## Classification of circRNA

### Circular intronic RNAs

In the splicing reaction, the circular intronic RNAs generate the lariat; therefore, the intronic regions are circularized and may avoid the debranching enzyme by the *DBR1* gene. An ideal circRNA is generated by trimming the 3′-linear end using an exonuclease. In back-splicing, a process that covalently closes distinct RNAs known as circRNAs, the 5′ donor splice site (SS) and the 3′ acceptor SS come together during canonical linear splicing to form a 5′–3′ splice junction (consensus sequence AG/GT). A 3′–5′ back-splicing junction with the unique consensus sequence GT/AG is produced when a donor SS interacts with an acceptor SS upstream (Zhang et al. 2013).

### Exonic circular RNAs

Exonic circular RNAs (EciRNAs) comprise most circRNAs and are composed entirely of exons. EciRNAs are mainly located in the nucleus and maintain intronic regions between the back-spliced exons. Over 80% of circRNAs are ecircRNAs, which are made up of one or more exons. EcircRNAs are produced by non-sequential back splicing in which covalent bonds form between a pre-mRNA downstream splice donor and an upstream splice acceptor (Barrett and Salzman 2016). Compared to single-exon circRNAs, exons in multiple-exon circRNAs are substantially longer. Exon length influences how effectively back-splicing performs, since larger exons are more likely to generate circular RNAs than shorter ones (Barrett et al. 2015). In ES, one or more exon sections are deleted with an upstream exon 3′ splice acceptor site. The circular structure of the nascent RNA transcript has two flanking introns that are alternatively spliced exons. After that, an EcircRNA is created by cutting these intronic sequences (Wang et al. 2017).

### Exon–intron circRNAs

Exon–intron circRNAs are circular in structure and contain both intron and exon regions, also known as EIciRNAs. The internal repeat sequences may be important for their formation, which may be associated with ecircRNAs (Meng et al. 2017). EIciRNAs are mostly located in the nucleus, interact with U1 snRNP, and then induce the transcription of parental genes (Li et al. 2015). The key mechanism by which the U1 snRNA acts may be the particular RNA-RNA interaction between EIciRNAs and U1 snRNA that allows EIciRNAs to induce transcription and cis functions with their parental genes. Further evidence for the beneficial effects of EIciRNAs on the transcription of their parental mRNAs comes from the possibility that downregulating the expression of EIciRNA levels might lower the expression level of their parental mRNA (Quan and Li 2018).

## Biogenesis of circular RNA

**Fig. 1:**
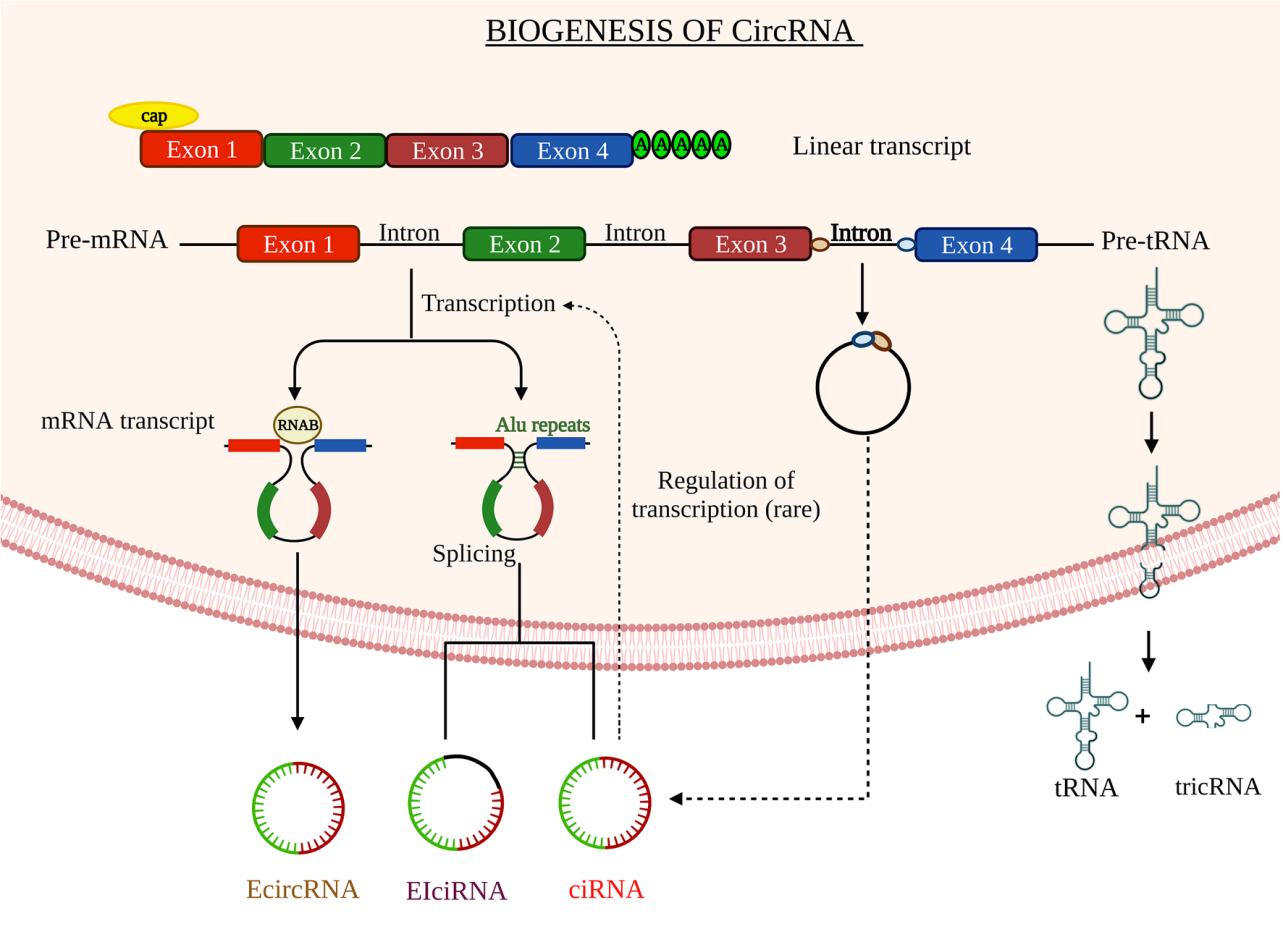
Biogenesis of circRNAs in breast cancer. EcircRNA is generated in three different methods: conventional splicing, lariat-driven circularization, intron-pairing driven circularization, intron cyclization, and formation of tricRNA. EIcirRNA is composed of exons and retained introns

Direct back-splicing occurs more frequently during the synthesis of exonic circRNA (ecircRNA) (Jeck and Sharpless 2014) than exon skipping (ES). The biogenesis and key regulatory functions of circRNAs are shown in Fig. 1. Most circRNAs originate from the 1–5 exons of protein-coding genes (Bolha et al. 2017). Most of the circRNAs originate from protein-coding exons, but occasionally they also originate from the 3’ and 5’ untranslated regions (3’UTR and 5’UTR), intronic and intergenic regions (Zhang et al. 2013). The spliceosome complex machinery regulates the process of alternative RNA splicing. It creates the circRNAs by back-spliced pre-mature mRNA, which joins with the donor of the upstream site 3’ and the acceptor of downstream splice region at the 5’ site. Back-splicing seems to be heavily controlled by canonical cis-splice acting site regulatory elements and the trans-acting splicing factors, much like canonical (linear) splicing. Back-splicing, which regulates the generation of circRNA, differs from linear splicing, which uses similar combinations of splicing regulatory elements and factors (Wang and Wang 2015). Therefore, these complexes are resistant to exonuleolytic destruction by RNase. Furthermore, ES creates a constrained lariat structure that may encourage cyclization. Currently, circRNAs are categorized into four groups based on their structure: circular intronic RNAs (ciRNAs), exon–intron cicRNAs (EiciRNAs), circular intronic RNAs (ecicRNAs), and tRNA intronic circular RNAs (tricRNAs) (Geng et al. 2020). So far, most of the circRNAs have been found in the cytoplasm, and > 80% of them are ecircRNAs (Jeck et al. 2013). The nucleus is the major location of cirRNAs and EiciRNas that promote transcription of genes and post translation process (Zhang et al. 2013). The first stage of circRNA biogenesis begins with the transcription of circRNA and pre-mRNA production by polymerase II. Second, cis and trans-regulatory cues can further influence the spliceosomal machinery that facilitates back-splicing. Intronic complementary sequences flanked the formatting of exons boundaries, core spliceosomal components, and additional regulatory RNA-binding proteins (RBPs). Third, circRNA turnover is crucial for regulating expression levels (Li et al. 2018). CircRNAs act as miRNA sponges, transcriptional and post-transcriptional regulators, parental gene expression controllers, and translator proteins in various disease states. Due to their presence in many biological samples, circRNAs can be used as biomarkers for the diagnosis and prognosis of neurological diseases, atherosclerotic vascular disease, and cancer (Mumtaz et al. 2020). CircRNA production is attributed to three primary processes: intron pairing-driven circularization, ES, and RBP-driven circularization.

### Intron pairing-driven circularization

In reverse complement, regions corresponding to neighboring introns serve as the basis for circulation. This creates an independent mechanism in the ES circulation. Reverse complementary regions can promote base-pairing between neighboring introns, promote hairpin structure formation, bring the 5’–3’ exon region into close contact, and induce head-to-tail splicing. Notably, this process involves reverse complementary matches (RCM) and the protein adenosine deaminase that acts on RNA (ADAR) (Yang et al. 2018). Substitution of adenosine residues in dsRNA molecules with creatinine molecules can induce unwinding and promote the production of circRNAs (Ivanov et al. 2015). Again, the presence of complementary regions in ALUs200 bps upstream or downstream of back splicing regions in non-circular transcribed regions suggests that splicing of intronic regions may cause the formation of exonic circRNAs (ecircRNAs) (Jeck et al. 2013).

### RBP-binding circularization

Introns and exons are joined by RBPs, which contribute to the formation of circRNAs. For instance, in the Drosophila model, the spliced regions in the muscle blind (MBL) selectively bind to the MBL-binding region inside introns bordering circMBL sequences. This process circumnavigates the 2^nd^ exon region of MBL to circularize and produce circMBL (Ashwal-Fluss et al. 2014). The interaction between MBL and circMBL regulates MBL protein levels. During the human epithelial-mesenchymal transition (EMT), a protein known as “quaking” (QKI) was found to promote the occurrence of circRNAs by binding with motifs inside the circRNA flanking intron position. Additionally, RBM20, an RBP, regulates various cardiac-specific gene-editing procedures. A mutation in *RMB20* contributes to cardiomyopathy by affecting titin-derived circRNA production (Khan et al. 2016).

## CircRNA biological functions

CircRNAs play essential roles in biological systems, including control of gene expression, development of miRNAs and endogenous RNA sponges, and functioning as biomarkers for disease diagnosis (Liu et al. 2019). Initially, the miRNA sponge binds to the miRNA, changes its biological behavior, and regulates the activity of miRNA target genes. Furthermore, miRNA regulates transcription or splicing in the nucleus. Among their functions, circRNAs counteract premature mRNA splicing to decrease linear mRNA and remove pre-mRNA to alter the processed mRNA. Further, circRNA interacts with RBPs and ribonucleoprotein complexes to inhibit their activity and act as “storage” components. In vitro, some circRNAs engage the roll loop amplification mechanisms that allow them to translate into proteins. However, the role of circRNAs in reverse transcription remains unclear (Liu et al. 2017). CircRNAs can bind to host genes at their site of synthesis triggering transcriptional pausing or termination by forming an RNA–DNA hybrid, which amplifies the truncated transcripts or skipped exon. CircRNAs can upregulate miRNAs, target mRNAs, and serve as miRNA sponges. CircRNAs with an internal ribosome entry site (IRES) can directly recruit ribosomes and initiate translation. CircRNAs including N6-methyladenosine (m6A) can be identified by YTH domain family protein 3 (YTHDF3). This prompts eIF4G2 to bind to initiate translation. Endogenous circRNAs suppress fat mass and obesity-associated proteins, leading to an increase in METTL3/14 in this process regulated by the YTH domain family protein 2 (YTHDF2) (Zhou et al. 2020). For instance, a circRNA produced from HIPK3 may regulate cell proliferation by sponging multiple miRNAs (Zheng et al. 2016).

**Fig. 2:**
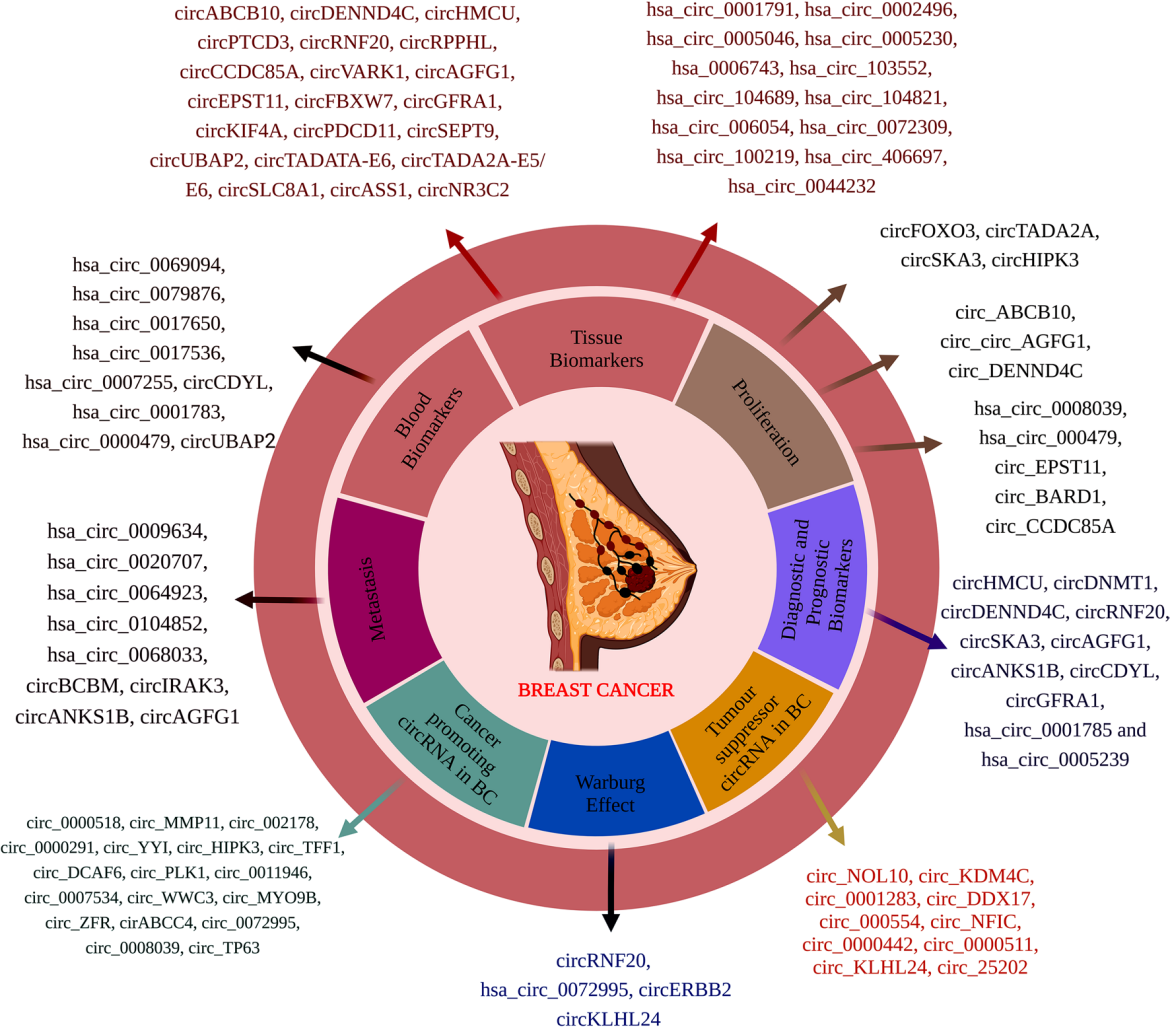
Role of circRNA in breast cancer as tissue biomarkers, blood biomarkers, metastasis, Warburg effect, diagnostic and prognostic biomarkers, cell proliferation, invasion and progression, cancer-promoting circRNAs, and tumor-suppressor circRNAs

The important role of circular RNAs is summarized in Fig. 2. Recently, circRNAs have been shown to encode biologically active proteins (Mei and Chen 2022). Furthermore, the Ago2-associated RNA-binding protein mediates the contact between circRNA and miRNA. CircRNAs can also alter the transcription of their parent genes, indicating a regulatory mechanism in gene transcription control through a particular RNA-RNA interaction. Circ-SHPRH and SHPRH-146aa both have high expression levels in healthy human brain tissue but are downregulated in glioblastoma-derived samples. When SHPRH-146aa is upregulated in glioblastoma cells of the U251 and U373 strains, these cells behave less malignantly and are less tumorigenic both in vitro and in vivo (Zhang et al. 2018).

## Involvement of circRNA in breast cancer onset and development

CircRNAs have been found to have crucial regulatory roles in cancer development and carcinogenesis (Chen et al. 2016; Loganathan and Doss 2023). CircRNAs help to discriminate between BC subtypes, offering a rapid suggestion regarding the therapy protocol to be followed with the patient and, thus, participating in improving the outcome. CircRNAs with prognostic significance may have therapeutic implications, as either silencing their expression or utilizing them as therapeutic targets may improve tumor prognosis (Dawoud et al. 2023). CircRNAs are stable because they do not have polyadenylated tails or 5′/3′ polarities (Li et al. 2015). The biogenesis of circular RNAs and their primary co-transcriptional nature set the stage for regulation of circular RNA transcription. The hundreds of circRNAs found in the mouse and human CNS are expressed several times over their linear isoforms (Rybak-Wolf et al. 2015). Through variable splicing, which entails the preferential selection of alternative splice sites while utilizing various splicing mechanisms and mRNA isomers, circRNAs can directly alter the transcription of the linear isoform. Additionally, circRNA regulates transcription by inducing DNA hypomethylation in the promoter region of the parental gene or by regulating the intronic enhancers. FECR1 circular RNA interacts with the *FLI1* promoter through extensive demethylation, which regulates the *FLI1* gene in the growth of breast cancer and promotes metastasis (Chen et al. 2018). The specific function of the circRNA-related ceRNA network in the development of breast cancer by thoroughly examining how the ceRNA network affects breast cancer (Zhao et al. 2019). A microarray study revealed that circTADA2A-E6 acts as a tumor suppressor with *SOCS3* as a downstream target gene. Also, another circRNA, circTADA-E5/E6, was downregulated in breast cancer (Xu et al. 2019). In another study, it was reported that Circ_000911 also acts as a tumor suppressor (Wang et al. 2018). Breast cancer was associated with an upregulation of circEPSTI1, a prognostic marker and a mediator in TNBC (Chen et al. 2018). In TNBC with lymph node metastases and progressive clinical stage, circANKS1B was increased. It inhibits cancer metastasis (Zeng et al. 2018). Diverse roles of circRNAs in promoting, regulating, and inhibiting breast cancer (He et al. 2021); or inhibiting drug resistance are illustrated in Table 1.

**Table 1:** A partial list of circRNAs involved in the promotion, growth inhibition, invasion, metastasis, and resistance to drugs in breast cancer

S. no	Functions	circRNAs
1	Promotes growth of breast cancer	CDR1-AS, circACAP2, circ_UBE2D2, hsa_circ_0000519, hsa_circ_0001982, hsa_circ_0007543, hsa_circ_0004771, hsa_circ_0008039, hsa_circ_0005230, hsa_circ_0052112, hsa_circ_001569, hsa_circ_0006528, hsa_circ_0056289, hsa_circ_069718, circGRAF1, circPLK1, circAGFG1, circ_0006528, circSEPT9
2	Inhibition of breast cancer	hsa_circ_0001098, hsa_circ_0087378, hsa_circ_0001184, hsa_circ_0025202, hsa_circ_0001283 and circ_ITCH
3	Promotes metastasis in breast cancer	hsa_circ_0011946, hsa_circ_0072995, circ_069718, circDENND4C, circ_TFF1, circANKS1B, circIRAK3, circHMCU, hsa_circ_0001944, and hsa_circ_21439
4	Inhibition of metastasis in breast cancer	hsa_circ_0072309, hsa_circ_103809, circTADA2A-E6, circBMPR2, circKDM4C, circASS1, hsa_circ_11783, circEHMT1 and circNFIC
5	Promotes resistant to drugs Adriamycin and Tamoxifen in breast cancer	circ_0006528 and hsa_circ_0025202
6	Inhibiting resistant to drugs adriamycin, tamoxifen, and monastrol in breast cancer	circKDM4C, circ_UBE2D2, and cirRNA_MTO1

## CircRNA in the regulation of parental genes

CircRNAs regulate their parental genes either directly or indirectly through the ceRNA-mediated mechanism. As an explanation, circGFRA1 may function covertly as a miR-34a sponge to regulate parental GFRA1 mRNA expression (He et al. 2017). Circ-ITCH restrains the expression of its parent mRNAs in breast cancer by sponging miR-17 and miR-214 ( Wang et al. 2019), hsa_circ_0001098 controls *BARD1* by sponging miR-3942 (Zhao et al. 2018), and hsa_circ_0052112 manages *ZNF83* by sponging miR-125a-5p (Zhang et al. 2018). It is interesting to note that some circRNAs such as circIRAK3 regulate *IRAK3* through a positive-feedback loop in which the target mRNA *FOXC1* attaches to the promoter of the parental gene *IRAK3* (Wu et al. 2018).

The cis effect of EIciRNA regulates the expression of parental gene (Li et al. 2015). CircRNAs directly regulate parental genes in cis; e.g., circ-*CNOT2* contains a start codon and circRNAs directly regulate parental genes in cis. For example, circ-CNOT2 contains a start codon, and circRNA binding can affect expression of linear genes, forcing them to use different start codons for translation, leading to some circRNAs interacting negatively with their parent mRNAs (Smid et al. 2019). However, EIciRNAs, a circRNA with introns “retained” between exons, have largely been found in the nucleus and linked with Pol II, U1 snRNPs, and other proteins (small nuclear ribonucleoprotein particles).

## Translation in breast cancer

Specifically, circRNAs are ncRNAs that have been detected using specialized bioinformatics tools that distinguish them from other types of RNA molecules such as linear RNAs. CircRNAs such as circ-CCDC66 induce cancer development and metastasis via a non-coding mechanism. A subset of circRNAs, however, possesses an open reading frame and is translated (Hsiao et al. 2017). An investigation on Drosophila, mouse, and rat revealed that a subset of circRNAs, including circ-Mb1, translate cap independently and generate proteins with specific protein domains that share a start codon with host RNA (Pamudurti et al. 2017). Another study found that splicing-dependent or cap-independent translation of circ-ZNF609 could result in a biologically active protein. Circ-FBXW7 is activated through an internal ribosome entry site and produces the FBXW7-185 protein, which suppresses cell growth and accelerates the cell cycle (Chen et al. 2019). However, the translation process of circRNAs among breast cancer patients remains unclear.

## CircRNAs are differently expressed in breast cancer

The expression pattern of circRNA in breast cancer in tissue and adjacent normal tissue was examined to identify the potential breast cancer-related dysregulated circRNAs. For instance, circRNF20 increased in breast cancer tissue compared with normal tissue and was derived from the 3^rd^ and 5^th^ exons of *RNF20* pre-mRNA. The prognostic study reveals that the survival rate decreases in breast cancer patients with increased expression of circRNF20. Therefore, cir-RNF20 may be identified as a prognostic marker. This might be explained as circRNF20 acts as a miRNA-487a sponge; therefore, cirRNF20/miR-487a targets hypoxia inducible factor-1α(HIF-1α) in breast cancer cells, indicating HIF-1α supports the transcription of HK2. CircDENND4C was significantly upregulated in breast cancer cells under hypoxia, which lowers both glycolysis and migration of breast cancer cells (Ren et al. 2019) and suggests that circRNF20 is crucial in determining the Warburg effect. These findings indicate a link between the axis circRNF20/miR-487a/HIF-1α and glycolysis in breast cancer (Cao et al. 2020).

In the early stages of breast cancer, hsa_circ_0006743 (circJMJD1C) and hsa_circ_0002496 (circAPPBP1) were overexpressed, indicating their potential use as biomarkers for early detection of breast cancer. Similarly, the variable expressions of hsa_circ_0005046 and hsa_circ_0001791 exhibit special relevance to the earlier clinical diagnosis of breast cancer (Rao et al. 2021). Other circRNAs with high levels of relevance for breast cancer diagnosis and prognosis include circ_HMCU, hsa_circ_0005230 (circRPPH1 015), hsa_circ_0087784 (circRNF20), hsa_circ_0005728 (circUBE2D2), and hsa_circ_0055478 (circPTCD3) (De Palma et al. 2022). Also, other circRNAs such as RERE, CREBBP, and CNOT2 were differentially expressed in primary breast cancer (Smid et al. 2019). In a microarray study on circRNA, 41 differentially regulated (two-fold) circRNAs were identified in a breast cancer patient in that 19 were upregulated and 22 were downregulated compared with healthy individuals (Yin et al. 2018). Another study revealed 1155 circRNAs with differential expression in breast cancer tissues, out of which 715 were upregulated and 440 were downregulated. Further, microarray studies on circRNA revealed that hsa_circ_103110, hsa_circ_104821, and hsa_circ_104689 were upregulated, and hsa_circ_006054, hsa_circ_406697, and hsa_circ_100219 were downregulated in breast cancer (Lü et al. 2017). A total of four genes (*LPP*, *MAML2*, *IGSF11*, and *Rev3l*) with protein-coding ability were linked in a study involving nursing rats using 1314 circRNAs during lactation stages in mammary glands of rats (Zhang et al. 2015). In 2006, Nair et al. created a Circ-Seq approach to detect circRNAs specific to breast tumors and then classified them into ER+, triple-negative breast cancer (TNBC: ER/PR/HER2-negative), and ERBB2 + (HER2 overexpression). Furthermore, in a case–control study, the typical mammary tissue samples from genotype-tissue expression, i.e., GTEx also reported different levels of circRNAs compared to the breast cancer tissue (Nair et al. 2016). Furthermore, ER+ circRNAs were inversely associated with the risk-of-relapse proliferation, indicating that these circRNAs may serve as cell proliferation indicators for overall breast cancer and subtypes (Lü et al. 2017). Some novel circRNAs were identified in breast cancer patients, such as circRNF20 (hsa_circ_0087784) and ciRS-7 (Chen et al. 2021), which were found to be among the top significantly upregulated circRNAs (Cao et al. 2020). One study showed that in early breast cancer, has_circ_0006743 (circJMJD1C) and has_circ_0002496 (circAPPBP1) were upregulated in cancerous tissues (Rao et al. 2021). Many circRNAs such as circANKS1B, circAGFG1, circIRAK3, circGFRA1, and circEPSTI1 play a role in breast cancer metastasis and chemoresistance by regulating the transcription of sub-types of RNA (miRNA and mRNA), in addition to monitoring the onset and progression of breast cancer.

Indeed, dysregulated expression of circRNAs including hsa_cir_0005046, hsa_circ_0001791, hsa_circ_0087784 (circRNF20), circHMCU, hsa_circ_0005728 (circUBE2D2), hsa_circ_0055478 (circPTCD3), and hsa_circ_0005230 (circRPPH1 015), has been reported with potential diagnostic and prognostic value for breast cancer (De Palma et al. 2022).

## CircRNAs as ceRNA or miRNA sponges in breast cancer

MiRNAs are crucial for post-transcriptional regulation of gene expression because they can bind to specific target sites in untranslated regions of mRNA. MiRNA sponge transcripts are known to compete for endogenous RNA. Specific circRNAs act as miRNA sponges in restraining cancer proliferation, metastasis, and invasion. Studies have shown that miRs such as miR-124-3p function as tumor suppressors in developing breast cancer (Wang et al. 2016). The discovery of CircHIPK3 was made through a luciferase screening assay, which revealed that the circRNA sponges to nine miRNAs with 18 potential binding sites. The researchers found that CircHIPK3 specifically binds to miR-124 and inhibits its activity, leading to increased proliferation of breast cancer cells (Zheng et al. 2016).

An upregulation of hsa_circ_0001982 circRNA was reported in breast cancer tissues and cell lines. The knockdown of hsa_circ_0001982 decreased the proliferation and invasion of breast cancer cells. By targeting miR-143, knockdown also induced apoptosis, providing insight into the pathogenesis and progression of breast cancer (Tang et al. 2017). Similarly, circRNA hsa_circ_0008717 (circABCB10) was found to be upregulated in breast cancer tissues. ABCB10 plays a key regulatory role in sponging miR-1271 in vitro in a loss-of-function assay, where knockdown of circ-ABCB10 reduced cell proliferation and increased apoptosis in breast cancer cells (Liang et al. 2017). Consequently, miR-591 silencing and overexpression of hexokinase 2 (HK2) mimicked the circ0069094 inhibition-mediated effects on cell growth, apoptosis, and glycolysis in breast cancer cells. Circ0069094 sponged miR-591 to increase HK2 expression. These results suggest that circ0069094 could be used as a therapeutic target and predictive biomarker for breast cancer since it increases cell viability and glycolysis (Xing et al. 2021). When circGFRA1 is knocked down, proliferation is inhibited, and apoptosis is activated, whereas circGFRA1 upregulation in breast cancers is associated with a poor survival rate in TNBC (He et al. 2017). The researchers speculate that circGFRA1 would act as a miR-34a sponge, regulating the ceRNA pathway to regulate GFRA1 production (He et al. 2017). Previous studies have provided a unique strategy in which circRNAs affect cancer progression by sequestering a variety of miRNAs involved in differentiation, proliferation, migration, and carcinogenesis.

## Signaling pathways that circRNAs use to regulate breast cancer onset and progression

Several studies have been conducted to determine the expression patterns and possible alterations of circRNA in breast cancer, as well as their possible association with intracellular key regulatory pathways of cell proliferation, migration, invasion, and differentiation. For instance, dysregulation of circRNAs has been involved in breast cancer formation by interacting with the Wnt/β-catenin cascade (Li et al. 2019). Previous studies showed that the classic Wnt/β-catenin signaling pathway inhibitor circ-ITCH was downregulated in TNBC and led to the inactivation of the Wnt/β-catenin signaling via sponging miR-17 and miR-214 (Lim et al. 2016). Additionally, cervical cancer cell lines exhibit low expression of circITCH, but its upregulation inhibits proliferation, migration, and invasion (Wang et al. 2019). Similarly, circ-ITCH suppresses CRC through the Wnt/β-catenin signaling pathway (Huang et al. 2015). Consequently, upregulation of circ-ITCH may be a successful strategy to inhibit breast cancer growth and progression, reduce the frequency of metastasis, and improve prognosis (Li et al. 2019). Similarly, recent research has shown that blocking the has_circ_0011946/RFC3 signaling pathway can reduce the ability of MCF-7 cells to invade and migrate (Zhou et al. 2018). In breast cancer patients, the miR-7 was downregulated compared with healthy breast tissue, suggesting it to be a potential tumor suppressor miRNA (Xin et al. 2017). Activation of miR-7 in invasive breast cancer cells reduced tumor cell proliferation, motility, and invasion (Kong et al. 2012). Interestingly, miR-7 is reported to be engaged in several signaling pathways related to cancer by directly suppressing the expression of crucial oncogenic proteins including FAK (Kong et al. 2012), KLF4 (Okuda et al. 2013), HER2D16 (Huynh and Jones 2014), REGγ (Shi et al. 2015), and SETDB1 (Zhang et al. 2014). The ciRS-7 and cir-miR-7 inhibitors are implicated in several cancer-related signaling pathways. Upregulation of miR-7 in MCF-7 and MDA-MB-231 reduced cell invasion and metastasis in breast cancer cell lines. Additionally, it prevents the growth of breast cancer stem cells (BCSC) via EMT by directly targeting and suppressing both *SETDB1* and *STAT3* genes. ciRS-7 may function as a miR-7 inhibitor to reduce the miR-7 inhibitory action on the STAT3 pathway (Zhang et al. 2014). By suppressing miR-7 target gene *EGFR*, CiRS-7 increases breast cancer cell proliferation by preventing MCF-7/HER2-16 complex-mediated cell migration.

In contrast, blockage of miR-7 and ciRS-7 may activate the EGFR pathway (Huynh and Jones 2014). These studies suggest that circRNA may activate or suppress signaling pathways associated with breast cancer growth and development. The PI3K/AKT/FOXO pathway is implicated in early initiation and spread of breast cancer (Smit et al. 2016). *FOXO3* was identified as a tumor suppressor gene with downregulation of circ-FOXO3 following cancer initiation due to increased Akt activity or loss of PTEN (Du et al. 2017). Yang et al. identified that Circ-Foxo3 might increase Foxo3 protein by binding to numerous miRNAs that share with *Foxo3* linear mRNA. It has been demonstrated that the PI3K/Akt/FOXO3a pathway is essential for regulating cell apoptosis (Yang et al. 2016).

The delicate hormonal balance, cell division, and death of breast tissue cells are affected by dysregulated biochemical processes associated with breast cancer (Velloso et al. 2017). A variety of cytokines, growth factors, and carcinogenic stimuli activate cellular transformation, tumor initiation, and development (Segatto et al. 2018). The MAPK signaling pathway is crucial in breast cancer development primarily through the following four primary routes; i) the JNK pathway, ii) the ERK pathway, iii) the p38/MAPK pathway, and iv) the ERK5/mitogen-activated protein kinase pathway. A loss-of-function mutation in the cJUN NH2-terminal kinase (JNK) signaling pathway switches on the *AP1* transcription factor and downregulates JNK signaling, promoting genomic instability and tumor development (Girnius et al. 2018). The major pathways leading to oncogenic ERK and chronic ERK-MAPK cascade activation involve mutationally activated Ras, B-Raf, EGFR, and HER-2 overexpression (Gee et al. 2001). Chemotherapy drugs such as paclitaxel inactivate the antiapoptotic MEK-ERK pathway and induce apoptosis (McDaid and Horwitz 2001). Post recurrence in TNBC patients, MAPK overexpression correlated with poor survival (Eralp et al. 2008; Bartholomeusz et al. 2012). Activating MAPK/JNK and p38MAPK pathway causes damage to mitochondria, which is connected with disease progression and prognosis of many cancers (Lai et al. 2021). p38 MAPK regulates cell fate by mediating cell survival or death depending on the type of stimulus and the specific cell type. It also critically regulates cell migration and invasion and is a target for counteracting tumor metastasis and cell survival (Koul et al. 2013). This pathway has been shown to promote healthy cell growth cycles, survival, and differentiation. The growth of malignant cancerous cells is regulated and accelerated via the MEK-MAPK pathway, promoting chemoresistance and antiapoptotic signaling (Drew et al. 2012). In converting breast cancer cells to BCSCs, numerous signal transduction pathways get dysregulated due to genetic and epigenetic alterations in MAP kinase, PI3K/Akt/NF-κB, TGF-β, Wnt/ β-catenin, Notch, hedgehog, and Hippo signaling. It limits the capacity of cancer stem cells for self-renewal, metastasis, and treatment resistance (CSCs) (Yousefnia et al. 2020). miR-7-5p has been shown to target Raf1, which in turn stimulates the MAPK/ERK signaling pathway. It was discovered that circ_0006528 enhances the expression of Raf1 and activates the MAPK/ERK pathway to promote breast cancer development, invasion, and migration (Gao et al. 2017).

In renal cell carcinoma, circHIAt1 can deregulate the miR-195-5p/29a-3p/29c-3p, which upregulated the CDC42 expression and enhanced cell migration and invasion of cancer cells (Wang et al. 2017). An upregulation of circTP63 in lung squamous cell carcinoma tissue led to an increase in cell proliferation. CircTP63 was found to bind to miR-873-3p, preventing the downregulation of FOXM1, a transcription factor involved in cell cycle progression. The dysregulation of circTP63/miR-873-3p/FOXM1 axis resulted in the acceleration of cell cycle progression and increased cell proliferation (Cheng et al. 2019). Bcl2 is a well-known antiapoptotic protein that can promote cancer cell survival by preventing programmed cell death. circUBAP2 and hsa_circ_0001892 are both circular RNAs that have been shown to inhibit apoptosis in osteosarcoma and breast cancer cells, respectively. These circRNAs do so by sponging up microRNAs (miRNAs) that would otherwise target and reduce the expression of antiapoptotic proteins such as Bcl2. By targeting miR-143, which would normally downregulate Bcl2 expression, circUBAP2 and hsa_circ_0001892 indirectly increase Bcl2 levels and thus promote cell survival (Zhong et al. 2018). During protein circRNA interactions, the circFoxo3 complex interacts with the cell cycle protein CDK2 and p21, thereby inhibiting cell cycle progression (Ng et al. 2018). circEIF3J and circPAIP2 belong to a specific type of circRNAs known as EIciRNAs. The RNA-RNA interactions between the U1 snRNA-EIciRNA complexes may interact with the RNA pol ll transcriptional complex in the parental gene to enhance gene expression (Li et al. 2015).

## CircRNA as breast cancer biomarkers

### CircRNA as indicators for diagnosis and prognosis

Breast cancer has complex and multifaceted molecular pathogenesis. As is widely known, most cancer types may be treated if detected early. Since the most widespread cancer screening methods and invasive or expensive methods include histology, magnetic resonance imaging, and chemotherapy, it is important to develop minimally invasive and cost-effective cancer detection methods. Additionally, prognostic assessment is important for early management of poor prognostic factors and extending the life expectancy of cancer patients.

Recent studies have revealed the following key characteristics of circRNAs: (1) diversity: high-throughput sequencing has identified > 100,000 circRNAs in human tissues (Glažar et al. 2014), (2) high expression: in fibroblasts, ~ 14% of genes transcribed are circRNAs; despite the low overall abundance of circRNAs, some exhibit substantially greater expression than linear RNAs (Jeck et al. 2013), (3) stability: circRNAs are more stable than linear RNA and may withstand destruction by RNA exonuclease or RNase R because they lack 5’–3’ polarity and a polyadenylated tail (Suzuki et al. 2006). Additionally, the typical half-life of mRNAs is 10 h. However, the circRNA half-life is more than 48 h, which has been observed in many species (Jeck and Sharpless 2014), (4) specificity: circRNA expression is cell type- and tissue-specific (Barrett and Salzman 2016), (5) universality: after linear RNAs, circRNAs are the most prevalent in human cells (Salzman et al. 2012), and (6) conservative: the circularization signal appears to be conserved across species in terms of evolution (Abou Haidar et al. 2014). Therefore, based on the literature, circRNAs are essential in cancer diagnosis and prognosis. For instance, in postoperative patients, the plasma level of hsa_circ_0001785 was lower compared to that in pre operative breast cancer patients (Yin et al. 2018), demonstrating its significance as a prognostic indicator.

### CircRNA tissue biomarkers in breast cancer

CircRNAs have undergone evolutionary conservation and exhibit tissue-specific expression. Because of their aberrant and differential expression, the identification of certain circRNAs may help to predict diagnosis, prognosis, and treatment response in patients with breast cancer. Common methods that have been employed to detect or validate circRNAs include RNA sequencing (RNA-seq) and microarray methods, digital droplet PCR, or RT-qPCR (Pandey et al. 2020). In breast cancer, some circRNAs have been considered oncogenic or tumor-suppressive biomarkers including circABCB10, circDENND4C, circHIPK3, circHMCU, circPTCD3, circRNF20, circRPPH1_015, hsa_circ_0001791, 0002496, 0005046, 0005230, 0006743, 103552, 104689, 104821, circCCDC85A, circLARP4, circVRK1, hsa_circ_006054, hsa_circ_0072309, hsa_circ_100219, and hsa_circ_406697. Similarly, circAGFG1, circEPSTI1, circFBXW7, circGFRA1, circKIF4A, circPDCD11, circSEPT9, circUBAP2, circTADATA-E6, circTADA2A-E5/E6, circSLC8A1, circASS1, circNR3C2, hsa_circ_0044232, and hsa_circ_0020397 were considered biomarkers in TNBC. Some biomarkers were detected in the cell line, i.e., TNBC vs. luminal, luminal/epithelial TNBC vs. mesenchymal TNBC cell lines (De Palma et al. 2022).

### CircRNA blood biomarkers in breast cancer

In addition to imaging studies, current breast cancer diagnostic techniques include cytological and histological methods, which often require invasive procedures. Liquid biopsy, a less invasive method, may avoid the challenges of surgical biopsy by reducing the need for solid tissues (De Rubis et al. 2019; Ignatiadis et al. 2021). By detecting and quantifying cancer-associated biomarkers in biofluids (such as blood, serum, urine, gastric juice, and breast milk), we can detect cancers (De Rubis et al. 2019). CircRNAs are exceptionally stable in circulation because of their circular and covalently closed conformation (Zhang et al. 2018), indicating that they might be useful to target circulating biomarkers in breast cancer. CircRNAs can circulate in extracellular vesicles (exosomes) or in the peripheral circulation as cell-free RNAs (Li et al. 2015; De Palma et al. 2020; Wen et al. 2021). Most studies have determined that serum or plasma samples derived from breast cancer patients contain higher concentrations of circRNAs than those from healthy individuals. Functional experimental research is needed to determine whether circRNAs play a role in breast cancer development.

Several circRNAs have been reported to be differentially expressed in breast cancer patients’ plasma samples, including hsa_circ_0069094, hsa_circ_0079876, hsa_circ_0017650, and hsa_circ_0017536 (Li et al. 2020). Additionally, hsa_circ_0007255 (Jia et al. 2020) and circCDYL (Liang et al. 2020) have also been reported to be differentially expressed in breast cancer. Some carcinogenic circRNAs were increased in breast cancer and thus may be represented as a new potential target for prognosis and treatment, e.g., hsa_circ_0000479 circEPST11 is sponged by miR4753 and miR-6809, regulating BCL11A and affecting TNBC proliferation and apoptosis (Chen et al. 2018). The hsa_circ_0001783 sponged by miR-200c_3p promotes the progression of breast cancer cells (Liu et al. 2019); hsa_circ _0001846 circ-UBAP2 sponges miRNA-661 and increases the expression of oncogene *MTA1* in TNBC (Wang et al. 2018). Some of the circRNAs including hsa_circ _0001982 (Tang et al. 2017), hsa_circ 0005230, hsa_circ _0005239 (Xu et al. 2018), hsa_circ _0007294 (circANKS1B) (Zeng et al. 2018), hsa_circ _0007534 (Song and Xiao 2018), hsa_circ _0008039 (Liu et al. 2018), and hsa_circ _0052112 were upregulated during breast cancer development. Both hsa circ _0005505 and circIRAK3 (Wu et al. 2018) increase metastasis; hsa circ _0006528 (Gao et al. 2017) induces adriamycin resistance in breast cancer cells. Dysregulated circRNA in breast cancer such as hsa circ _0008717 (circ-ABCB10) contributes to the proliferation by sponging miR-1271 (Liang et al. 2017). Similarly, hsa_circ_0011946 has been identified as a novel circRNA that is upregulated in breast cancer tissues compared to adjacent normal tissues, and it has been suggested to promote breast cancer cell proliferation by modulating the expression of several genes involved in cell cycle regulation and DNA replication (Zhou et al. 2018).

The study by Liang et al. (2017) reported that hsa_circ_0008717 (circ-ABCB10) was upregulated in breast cancer tissues and cell lines and promoted breast cancer cell proliferation and invasion by sponging miR-1271. This study showed that knockdown of hsa_circ_0008717 inhibited breast cancer cell proliferation and induced apoptosis in vitro and suppressed tumor growth in vivo, suggesting its potential as a therapeutic target in breast cancer (Liang et al. 2017). Similarly, the study by Zhou et al. (2018) reported that hsa_circ_0011946 was upregulated in breast cancer tissues and cell lines and promoted breast cancer cell proliferation and invasion by sponging miR-1236. The authors further showed that knockdown of hsa_circ_0011946 inhibited breast cancer cell proliferation and induced apoptosis in vitro, and suppressed tumor growth in vivo, suggesting its potential as a therapeutic target in breast cancer.

The miR-30c-2-3P is associated with has_circ_0072995 and enhances the migration and invasion of breast cancer cells. Breast cancerous cells have the same amounts of Hsacirc 0072995 in their nucleus and cytoplasm, which regulates invasion and migration (Zhang et al. 2018). However, the downregulation of hsa_circ_0052112 interferes with the migration and invasion of breast cancer cells (Zhang et al. 2018). circ-ITCH acts as a tumor suppressor by sponging miR-214 (which overexpresses in TNBC) and promotes cell proliferation and migration of cancerous cells. These results suggested circ-ITCH to be a potential prognostic biomarker for TNBC patients (Wang et al. 2019). By deactivating the wnt/β-catenin signaling pathway, Circ-ITCH interacts with miR-214 and miR-17 in TNBC along with upregulated linear isoform of ITCH (Raut et al. 2022). In breast cancer patients, the low-level expression of circ_0000911 correlated with poor prognosis, and it was further noticed that overexpression of miR-449a sponges circ_000911 with Notch1, regulating NF-κB signalling, and induces the apoptosis and suppress the proliferation, invasion, and metastasis of breast cancer cells (Abdollahzadeh et al. 2019). Exposure to tetrachlorodibenzo-p-dioxin (TCDD) increased the level of has_circ_0001098, which inhibited breast cancer development via the miR-3942-3p/BARD1 axis. These new findings on the TCDD-circRNA-miRNA-mRNA axis could open up new treatment options for breast cancer (Zhao et al. 2018). The circRNA-MTO1 (hsa circ 007874), on the other hand, was associated with elevated expression levels in monastrol-resistant cells and decreased cell viability, enhanced monastrol-induced cell cytotoxicity, and reversal of monastrol resistance. In breast cancer cells, circRNA-MTO1 regulates cell viability and monastrol resistance. Hsa circ 0087378 appears to be downregulated in ER+ breast cancer, making it an attractive potential target.

## Exosomal circRNA in breast cancer

Exosomes are 30–100 nm microvesicles released by most cells with a wide range of functional molecules including proteins and RNAs. In prior investigations on breast cancer, most circRNAs were found in the cells or tissue RNA samples, while only a few were found in plasma. In 2015, it was revealed that exosomes contain many circRNAs (Lener et al. 2015).

Exosomes containing over 1000 circRNAs have been discovered in human serum. Exosomes play a critical role in intercellular communication as they act as nucleic acid transporters, delivering genetic information to recipient cells both near and far (Tetta et al. 2013; Milane et al. 2015). Therefore, they may serve as biomarkers for the determination of cancer cell proliferation (Nair et al. 2016). Exosomes and cell expression profile of the MDA-MD-231 cell line showed altered gene expression. There were five circRNAs found to be upregulated in exosomes derived from highly aggressive cells and metastatic tumors in breast cancer patients. These circRNAs were hsa_circ_0009634, hsa_circ_0020707, hsa_circ_0064923, hsa_circ_0104852, and hsa_circ_0087064 (Wang et al. 2019). A study by Lin et al. demonstrated the presence of nine circRNAs in plasma extracellular vesicles that were upregulated. These circRNAs are named hsa_circ_0005552, hsa_circ_0007177, hsa_circ_0002190, hsa_circ_0001439, hsa_circ_0000642, hsa_circ_0006404, hsa_circ_0000267, hsa_circ_0001073, and hsa_circ_0001417 (Lin et al. 2021). There had been a handful of investigations on exosomal circRNA in breast cancer. The current and emerging knowledge of exosomal circRNA in breast cancer could offer options for disease alternative therapy in the near future.

### Exosomal circRNA in chemoresistance/radioresistance

The results of some studies looking at the expression of circRNAs in chemoresistant breast cancer have suggested that they might play a role in breast cancer metastasis or transformation, e.g*.,* exosome-transmitted circHIPK3, which induces trastuzumab chemoresistance in drug-sensitive breast cancer cells (Zhang et al. 2021). Although there have been a handful of studies on circRNAs and chemoradiation resistance in cancer, circRNAs can be used as novel biomarkers to assess the efficacy of chemotherapy and determine the likelihood of recurrence in drug-resistance. These studies showed that in vitro transduction of circ-UBE2D2 via exosomes can increase resistance to tamoxifen in breast cancer. Recently, the expression of circ-UBE2D2 (MiR-200a-3p is the target of circ-UBE2D2) was reported to be 20-fold higher in MCF-7/TAM-R-Exo as compared with MCF-7/Par-Exo (Nie et al. 2020).

### CircRNA association with EMT

The development of embryonic organs, among other physiological and pathological processes, depends on EMT (Sakabe et al. 2006; Matoba et al. 2017), wound healing, and tumor metastasis (Feldkoren et al. 2017; Nishiyama et al. 2018). The majority of patients die due to tumor metastatic complications; EMT is crucial at the initiation of cancer metastasis, and proliferation, so it might be a potential new therapeutic target. During EMT, epithelial cells lose cell-to-cell adhesion and polarity while gaining motility by acquiring migration and invasion characteristics to change them into mesenchymal stem cells. The EMT-induced transcription factors (EMT-TFs) in cancer are complicated because these proteins frequently play additional tissue-specific, non-redundant activities. For instance, it has been proven that SNAIL (snail1) is a zinc finger transcription factor that promotes EMT-initiating breast cancer metastasis (Tran et al. 2014; Brabletz et al. 2018).

During the EMT process, circRNA adsorbs the miRNA and diminishes miRNA repression on EMT-related genes. Epithelial cells lose their ability to adhere to other cells and maintain cell polarity. EMT results in primary tumor cells migrating into the lymphatic or circulatory systems and forming secondary metastatic deposits (Shang et al. 2019). For instance, circRNAs associated with the TGF-β/smad pathway can enhance the EMT process. In TNBC, upregulation of circANKS1B impairs prognosis and encourages breast cancer invasion and metastasis. The miR-148a-3p interacts with miR-152-3p increasing upstream transcription factor1 (USF1) expression mediated by Circanks1B (Zeng et al. 2018). In TNBC tissues, increased USF1 interacts with TGF-1 to increase the expression of vimentin, p-Smad2, and p-Smad3 while decreasing the expression of E-cad (E-cadherin). A positive feedback regulatory loop involving circANKS1B, USF1, and ESRP1 formed splicing epithelial splicing regulatory protein 1 (ESRP1) by USF1 (Zeng et al. 2018). Several investigations have shown that splicing factors (SFs) affect circRNA formation and EMT progression (Ashwal-Fluss et al. 2014; Yu et al. 2017). In the EMT model of breast cancer cells, circSCYL2 and its target gene *OSR1,* which interacts with the TGF pathway, were dysregulated, but upregulating circSCYL2 in breast cancer cells inhibited cell migration and invasion. Future studies on circRNAs associated with EMT development might develop valid diagnostic and prognostic markers for breast cancer and novel therapeutic approaches (Yuan et al. 2020).

### CircRNA invasion, metastasis, and Warburg effect

The extreme outcome of cancer growth is metastasis, which makes malignant tumors difficult to treat. The most common cause of death in patients is the spread of metastatic cells to other organs. Therefore, understanding the molecular processes of tumor metastasis is crucial for better disease management. CircRNAs primarily affect tumor metastasis through protein binding and miRNA-sponging mechanisms. CircRNAs and other ncRNAs have been shown to regulate metastasis in several cancer types, and dysregulated circRNA expression varies between original tumors, metastatic disease, and healthy tissues. The cancer cells often exhibit altered glucose metabolism, which is commonly referred to as the Warburg effect. Under aerobic conditions, cells typically use glucose to produce ATP through oxidative phosphorylation (OXPHOS) and only enter glycolysis under anoxic conditions. However, cancer cells choose glycolysis even in an environment with normal oxygen levels. The Warburg effect is the name given to this phenomenon since Otto Warburg first described it in the 1920s (Warburg 1956).

Interestingly, several circRNAs are implicated in this phenomenon (Yu et al. 2019), e.g., circAmotl1 (Zeng et al. 2017; Yang et al. 2017) and circDENND4C (Liang et al. 2017) stimulate glycolysis in breast cancer. This effect can be attributed to the role of circRNAs in glycolysis through stimulating transporters and metabolic enzymes. MiR-124 is sponged by circHIPK3, which inhibits the expression of several glycolysis-related enzymes and glucose transporters (Zhao et al. 2017; Zheng et al. 2016). According to a previous study, inhibition of circHIPK3, which is prevalent in pancreatic islets, reduced the expression of the *SLC2A2* gene (solute carrier superfamily of sodium-glucose cotransporter), which encodes *GLUT2* (glucose transporter) (Stoll et al. 2018). Studies have shown that circ-Amotl1 and another circRNA may physically bind to PDK1 and AKT1 and move them into the nucleus to prevent apoptosis (Zeng et al. 2017). In tumor tissues, these circRNAs are thought to exert the Warburg effect by regulating transcription factors (TFs) in the Wnt/β-catenin pathway, including MCT1 and PDK1. In a hypoxic environment, CircDENND4C, which has been shown to be upregulated by HIF-1 in breast cancer cells, actively stimulates cell proliferation (Liang et al. 2017) to achieve the Warburg effect by providing sufficient energy for rapidly growing cancer cells.c-Myc, one of the most significant oncogenic TFs, can initiate the Warburg effect. Ectopic c-myc and HIF-1 may act synergistically to increase the concentration of the glycolytic enzymes GLUT1, HK2, and PDK1 (Kim et al. 2007; Li and Zhang 2016). PI3K/Akt, RAS, STAT3, and Wnt/β-catenin pathways associated with glycolysis, lipid metabolism, and the Warburg effect are regulated by circRNAs. For instance, circRNA_0046367 may accelerate tumor progression by increasing fatty acid β-oxidation (FAO) (Yu et al. 2019). In triple-negative breast cancer, CircGFRA1 targets miR34a as a ceRNA to release GFRA1, which can activate autophagy (He et al. 2017). CircRNA VRK1 was negatively correlated to breast cancer stem cells (Yan et al. 2017). CircBIRC6 participates in the Warburg effect by preventing cancerous cell differentiation. FAO appears to be essential for the renewal of hematopoietic stem cells and cancer stem cells (Samudio et al. 2010; Ito et al. 2012). Therefore, it can be expected that circRNAs belonging to FAO may participate in this process. A better understanding of the circRNF20/miR-487a/HIF-1/HK2 axis in breast cancer progression and the Warburg effect may provide new insights into breast cancer (Cao et al. 2020).

### Tumor-suppressive circRNA in breast cancer

CircRNAs with tumor-suppressive properties play an important role in tumorigenesis and metastasis, making them targets for cancer therapy. In accordance with the publications, we outline the functions and processes of circRNAs that play tumor suppressor roles in breast CSCs. Several tumor suppressor circRNAs have been shown to play an important role in attenuating tumor growth via the upregulation of tumor suppressor genes (Huang et al. 2021a). Overexpression of tumor suppressor genes reduces the ability of cancer cells to proliferate and form colonies. Indeed, tumor-suppressive circRNA upregulation can absorb tumor-promoting miRNAs, accelerating programmed cell death (PCD) and inhibiting EMT (Heerboth et al. 2015; Brabletz et al. 2018). In contrast, breast cancer and TNBC tissues and cells showed detectable downregulation of tumor-suppressive circRNAs. As a result, a mesenchymal phenotype and stemness characteristics are acquired, and epithelioid cancer cells gradually lose their polarity, solid intracellular connections, and resistance to treatment (De Palma et al. 2022). Invasive breast cancer and distant metastasis are negatively correlated with expression of tumor-suppressive circRNAs. Poor prognosis, large tumors, advanced TNM staging, and lymph node metastases were all associated with reduced circRNA expression in breast cancer patients. Many tumor suppressor circRNAs have been identified in breast cancer and TNBC, including circ_NOL10, circ_KDM4C, circ_0001283, circ_DDX17, circ_000554, circ_NFIC, 0000442, circ_0000511, circ_KLHL24, circ_0025202, circ_NR3C2, circ_TADA2As, and circ_FBXW7 (Huang et al. 2021a).

CircRNA VRK1 negatively correlated with breast CSCs suppressing breast CSCs. circ_000911, hsa_circ_0072309, and circASS1 are tumor-suppressive circRNAs in breast cancer with potential as therapeutic targets. In other study, hsa_circ_0072309 suppresses the overexpression of miR492 activity, which attenuates breast cancer progression (Yan et al. 2019). Furthermore, circASS1 stops the onset and spread of breast cancer by activating the miR-4443/ASS1 axis (Hou et al. 2019).

## CircRNA databases

Several databases and online resources have been developed to provide information on circRNAs and regulatory networks. A partial list of these databases is presented in Table 2. These online databases made it feasible to analyze the differential expression of circRNA between diseased vs*.* normal tissues and/or cells, which eventually helped the scientific community learn and understand the functioning of circRNAs in diseases like breast cancer.

**Table 2:** Different online databases on circRNAs with their web address

Database	Description	URL	References
circBase	It is an extensive repository for publicly available circRNA datasets	http://www.circbase.org/	(Glažar et al. 2014)
circ2traits	A database of human circRNAs linked to traits or diseases	http://ww3.gyanxet-beta.com/circdb/	(Ghosal et al. 2013)
CircNet 2.0	A circular RNA database was developed using transcriptome sequencing data and for circRNAs regulating networks for cancers	https://awi.cuhk.edu.cn/~CircNet/php/index.php	(Chen et al. 2022)
circInteractome	Circular RNA interactome (circRNA exploration, siRNA, and primer design, as well as circRNA interaction with proteins or miRNAs)	https://circinteractome.nia.nih.gov/	(Dudekula et al. 2016)
CIRCpedia	circRNA alternate splicing and alternative back-splicing annotation	http://yang-laboratory.com/circpedia/	(Dong et al. 2018)
circRNADb	circular RNA DataBase V1.0 (a comprehensive database of human circular RNAs with annotations for protein coding a database of data about cancer)	http://reprod.njmu.edu.cn/cgi-bin/circrnadb/circRNADb.php	(Chen et al. 2016)
CSCD	Cancer-specific circRNA database	http://gb.whu.edu.cn/CSCD/	(Xia et al. 2018)
TSCD	Tissue-specific circRNA database	http://gb.whu.edu.cn/TSCD/	(Xia et al. 2017)
circAtlas 2.0	Annotation of circRNAs using an integrated network of circRNAs, mRNAs, miRNAs, and RBP	http://circatlas.biols.ac.cn/	(Wu et al. 2020)
circbank	An extensive library of human circRNAs that uses a unique name scheme for circRNAs based on their host genes	http://www.circbank.cn/	(Liu et al. 2019)
TransCirc	It is an online database used in prediction of circRNAs translation and potential circRNAs encoded peptides, the data can be retrieved and analysed	https://www.biosino.org/transcirc/	(Huang et al. 2021a)
CircR2Disease V2.0	It is an comprehensive webtool to understand the relationship between circRNAs and diseases in various species. It also uselful in explore roles of dysregulated circRNAs in different diseases and post-transcriptional regulatory function	http://bioinfo.snnu.edu.cn/CircR2Disease_v2.0/	(Fan et al. 2021)

## Challenges in studying the circRNAs and future perspectives

CircRNAs are stimulatory molecules in the regulation and transformation of breast cancer cells, and many unknown factors are involved in their biological regulatory mechanisms in normal and pathological states. Generally, circRNAs accumulate in the cell cytoplasm, but the mechanism of their nuclear export is largely unknown. Several studies have shown that RBP-mediated selective transport or N6-methyladenosine (m6A) modification promotes circRNA cytoplasmic export (Huang et al. 2021b; Ma et al. 2021). With respect to the knowledge of circRNAs, many questions remain unanswered, such as what defines long and short circRNAs, how the pathways are regulated for different length circRNAs, and the extra-nuclear mechanisms of circRNAs’ export and the nature of these unknown features. Collectively, understanding these questions will help us understand the biogenesis and functions of circRNAs. Synthetic circRNAs have been used for in vivo tumor targeting. In comparison to endogenous circRNAs, synthetic circRNAs lack m6A modifications. In this regard, it is necessary to look at the ways to reduce immunogenicity by chemically modifying synthetic circRNAs and covering them with RBPs (Tao et al. 2021)﻿. Regarding the functions of circRNAs in cancer, knowledge will improve as we better understand the mechanisms controlling circRNA fate, the downstream effects of circRNA regulatory networks, and the clinical significance of circRNAs in cancer. The circRNA-based drugs, prognosis, and diagnostics promise a new frontier in cancer treatment. However, there are challenges in targeting circRNAs, including off-target effects and tissue-specific expression patterns. Some methods to target circRNAs include RNA interference (RNAi) and the CRISPR/Cas13 system. Although RNAi can effectively attenuate circRNA expression, it can also have off-target effects and cause changes in the expression of linear mRNA levels. The CRISPR/Cas13 system has shown promise as a highly specific and efficient method for targeting circRNAs with a low mismatch tolerance and high specificity. Additionally, nanoparticle delivery systems can be explored to achieve site-specific delivery of circRNA-targeted therapies. Gold nanoparticles have shown promise as a delivery vehicle, but their toxicity remains a significant concern. Alternatively, lipid nanoparticle (LNP)-siRNA systems, approved for the treatment of hereditary transthyretin amyloidosis, may be a possible alternative for targeting breast cancer cells with improved therapeutic index. However, further research is needed to fully understand the potential of circRNAs to act as diagnostic markers, prognostic predictors, and therapeutic targets in cancer therapy, and to develop effective and safe delivery methods for circRNA-based therapies.

## Conclusions

Circular RNAs (circRNAs) are a group of ncRNAs that have gained recognition for their function in many biological processes including cancer. They are distinguished by a closed circular structure that improves their stability and resistance to degradation, allowing them to persist in biological samples such as blood, saliva, and urine. Although the roles of many circRNAs are still unclear, some circRNAs have been found to be identified as key participants in cancer biology. Notably, some circRNAs have been shown to promote the proliferation and survival of cancer cells, while others have been shown to inhibit cancer cell growth. Furthermore, some circRNAs have been linked to chemotherapy resistance in cancer cells, emphasizing their importance in cancer management. Recent advances in circRNA research have increased interest in using these molecules as diagnostic markers, prognostic predictors, and therapeutic targets for cancer. Future studies aimed at elucidating the specific roles of circRNAs in cancer may lead to the development of novel therapeutic strategies that target these molecules and improve cancer treatment outcomes.

## Data Availability

The research data associated with a paper is available in the manuscript or will be available on requests to the corresponding author.
